# Characterisation of T cell receptor repertoires in coeliac disease

**DOI:** 10.1136/jcp-2022-208541

**Published:** 2022-12-15

**Authors:** Lik Wee Lee, Shahin Shafiani, Beryl Crossley, Ryan O Emerson, David Williamson, Anna Bunin, Justin Vargas, Arnold S Han, Ian M Kaplan, Peter H R Green, Ilan Kirsch, Govind Bhagat

**Affiliations:** 1 Computational Biology and Translational Medicine, Adaptive Biotechnologies Corp, Seattle, Washington, USA; 2 Department of Medicine, Celiac Disease Center, Columbia University Irving Medical Center, New York, New York, USA; 3 Department of Pathology and Cell Biology and Department of Medicine, Celiac Disease Center, Columbia University Irving Medical Center, New York, New York, USA

**Keywords:** celiac disease, inflammation, gastrointestinal diseases, autoimmunity

## Abstract

**Aims:**

Characterise T-cell receptor gene (TR) repertoires of small intestinal T cells of patients with newly diagnosed (active) coeliac disease (ACD), refractory CD type I (RCD I) and patients with CD on a gluten-free diet (GFD).

**Methods:**

Next-generation sequencing of complementarity-determining region 3 (CDR3) of rearranged T cell receptor β (TRB) and γ (TRG) genes was performed using DNA extracted from intraepithelial cell (IEC) and lamina propria cell (LPC) fractions and a small subset of peripheral blood mononuclear cell (PBMC) samples obtained from CD and non-CD (control) patients. Several parameters were assessed, including relative abundance and enrichment.

**Results:**

TRB and TRG repertoires of CD IEC and LPC samples demonstrated lower clonality but higher frequency of rearranged TRs compared with controls. No CD-related differences were detected in the limited number of PBMC samples. Previously published LP gliadin-specific TRB sequences were more frequently detected in LPC samples from patients with CD compared with non-CD controls. TRG repertoires of IECs from both ACD and GFD patients demonstrated increased abundance of certain CDR3 amino acid (AA) motifs compared with controls, which were encoded by multiple nucleotide variants, including one motif that was enriched in duodenal IECs versus the PBMCs of CD patients.

**Conclusions:**

Small intestinal TRB and TRG repertoires of patients with CD are more diverse than individuals without CD, likely due to mucosal recruitment and accumulation of T cells because of protracted inflammation. Enrichment of the unique TRG CDR3 AA sequence in the mucosa of patients with CD may suggest disease-associated changes in the TCRγδ IE lymphocyte (IEL) landscape.

**WHAT IS ALREADY KNOWN ON THIS TOPIC**

- Studies aimed at delineating coeliac disease (CD) pathogenesis have mostly focused on characterising small intestinal and peripheral blood TR repertoires of enriched, gliadin-specific CD4^+^ T cells.

**WHAT THIS STUDY ADDS**

- Analysis of the overall TR repertoires in the small intestinal IE and LP compartments of CD patients, in an unbiased manner, revealed a higher T cell density and lower clonality in both compartments in untreated (active) CD and refractory CD type I, and confirmed the presence of public gliadin-specific TRB sequences in the LP in a high proportion of CD cases. Certain public TCRγδ T cell-derived TRG CDR3 AA motifs, encoded by multiple nucleotide variants, were found to be more abundant in the IE compartment of CD patients compared with controls.

**HOW THIS STUDY MIGHT AFFECT RESEARCH, PRACTICE OR POLICY**

- The identification of CD-specific public TRG sequence motifs may help uncover yet unknown facets of CD biology and assist in the development of novel diagnostic biomarkers.

## Introduction

Coeliac disease (CD) is an immune-mediated malabsorption syndrome occurring in genetically susceptible individuals intolerant to dietary gluten.^1^ The pathogenesis of CD involves the generation of effector innate and adaptive immune responses.^1^ The latter involves activation of gliadin-specific CD4^+^ TCRαβ T cells within regional lymphoid tissue, with trafficking of effector memory gliadin responsive CD4^+^ T cells to the lamina propria (LP), and concomitant stimulation of humoral immune responses directed towards tissue transglutaminase type 2 and deamidated gliadin. Activation of innate intraepithelial (IE) CD8^+^ TCRαβ T cells has been shown to play a role in epithelial damage.^2^ Recruitment of TCRγδ T cells to the IE compartment is also a characteristic feature of CD.^3^ Indeed, IE TCRγδ T cells are increased in all stages of CD, with some reports suggesting immunoregulatory roles for a subset.^4–9^

Investigations of specific innate and adaptive immune cells have contributed to a better understanding of cellular networks and signalling pathway that are dysregulated in CD. However, the breadth of and temporal changes in immune responses, both locally and systemically, remain to be clarified. Analysis of the immune receptor repertoires to determine changes in B and T cell diversity in different tissue compartments has enabled dissection of antigen-specific and non-specific immune responses in an unbiased manner,^10 11^ and for T cell-mediated diseases, presence of clonal T cell expansions have suggested the presence of disease triggering antigens.^12^ Studies of LP CD4^+^ TCRαβ T cell repertoires in CD have helped shed light on the adaptive immune response to gluten in the small intestinal mucosa,^13^ but our knowledge of alterations in IE innate CD8^+^ TCRαβ and TCRγδ T cell repertoires is more limited.^14 15^

A variety of methods have been used to study T cell repertoires in the past including V-region-specific monoclonal antibody-based flow cytometry and TR spectratyping (immunoscope analysis), but these modalities had limited resolution.^10^ The introduction of next-generation sequencing (NGS) has enabled comprehensive characterisation of the TRB and TRG repertoires. The current study used an NGS-based method to sequence rearranged TRB and TRG CDR3 regions of lymphocytes from different small intestinal mucosal compartments and a limited number of peripheral blood mononuclear cell (PBMC) samples from patients with different clinical presentations or phases of CD and controls, enabling quantitative evaluation of disease-associated changes in the TR repertoires.^16 17^

## Materials and methods

### Sample acquisition and isolation of cellular fractions

Altogether 83 discrete samples from 30 patients were analysed, 22 with serologically confirmed CD and 8 with non-small intestinal disorders, serving as controls. The different types of samples analysed were as follows: total intraepithelial cells (IECs) from all 30 patients, total LP cells (LPCs) from 27 patients, PBMCs from 6 patients and 20 flow-sorted samples of different IEC and LPC T-cell subsets. The details of all the samples evaluated are provided in table 1 and online table S1. Information about patient classification and clinical, pathology and laboratory data are provided in online supplemental methods and table S1. Lymphocyte fractions were isolated from small intestinal biopsies as previously described,^18 19^ pertinent details are provided in online supplemental methods. Aliquots of 500 000 IECs and LPCs were suspended in dimethyl sulfoxide (DMSO)/fetal bovine serum (FBS) and stored in liquid nitrogen. One aliquot of each cellular fraction was used for sequencing. The PBMC isolation method is described in online supplemental methods. One PBMC aliquot, comprising 1,000,000 cells, was used for sequencing.

10.1136/jcp-2022-208541.supp2 Supplementary data

10.1136/jcp-2022-208541.supp1 Supplementary data

**Table 1:** Details of samples used in the study

Donor status	TRB locus					TRG locus					
	IEC	LPC	PBMC	CD8^+^ TCRαβ^+^ IEC	CD4^+^ TCRαβ^+^ LPC	IEC	LPC	PBMC	CD8^+^ TCRαβ^+^ IEC	CD4^+^ TCRαβ^+^ LPC	TCRγδ^+^ IEC
Active coeliac disease (CD)	10	8	2	4	2	10	8	2	4	2	2
Refractory CD type 1	3	3	0	0	0	2	2	0	0	0	0
CD—gluten-free diet	8	7	2	2	2	9	8	2	2	2	2
Controls	6	6	2	2	2	8	8	2	2	2	2
Total	27	24	6	8	6	29	26	6	8	6	6

Table provides a breakdown by donor type, including control and CD donors categorised by CD status, the number of actual samples by tissue type subjected to bulk population analysis, and IEC and LPC samples that were subjected to cell sorting including the sorted cell type.

IEC, intraepithelial cell; LPC, lamina propria cell; PBMC, peripheral blood mononuclear cell.

### Fluorescence-activated cell sorting

Cell sorting was performed using a BD FACSAria II instrument, and FACSDiva V.6.1.3 software was used for data acquisition and analysis (BD Biosciences, New Jersey, USA). Three T cell populations were sorted from a subset of cases: TCRγδ (two samples each of active CD [ACD], gluten-free diet [GFD] and controls), CD8^+^ TCRαβ T cells from IEC fractions (four ACD samples and two samples each of GFD and controls), and CD4^+^ TCRαβ T cells from LPC fractions (two samples each of ACD, GFD and controls), as listed in table 1. Further details on sorting strategy are provided in online supplemental methods and the number of cells sorted for the different T cell lineages from the IEC and LPC samples are included in online table S2.

10.1136/jcp-2022-208541.supp3 Supplementary data

### TR sequencing

Genomic DNA was extracted from aliquots of frozen total IEC, LPC and PBMC fractions as well as sorted T cell populations using the Qiagen DNeasy blood extraction Kit (Qiagen, Gaithersburg, Maryland, USA) and sequencing of rearranged TRB and TRG loci was performed (immunoSEQ, Adaptive Biotechnologies, Washington, USA) as reported previously^16 17^ and described in online supplemental methods. The TR sequencing data of all the samples are available from the Adaptive Biotechnologies immuneACCESS repository: https://doi.org/10.21417/LWL2022JCP.^20^

### TR repertoire analysis

In order to obtain a representation of the total TRB and TRG repertoires for each unsorted (and sorted) sample, the nucleotide sequences representing the CDR3 regions were translated into their corresponding amino acid (AA) sequences. A number of parameters of the individual repertoires from each sample were analysed, including the total number of sequences identified, the overall clonality,^17^ the frequency distribution of the individual sequences and the degree of convergent recombination (the number of nucleotide sequences generating the same AA sequence). The individual samples were compared for co-occurrence of sequences and a differential abundance algorithm was applied to identify AA sequences that were present in multiple samples, in order to assess for the presence of potential disease-associated sequences. These methods have been published previously.^21 22^ We also investigated the presence of known gliadin-specific TRB clonotypes in LPC and available PBMC samples by querying the sequences against a set of published CD LP TRB sequences derived from HLA-DQ2.5 gliadin tetramer-sorted CD4^+^ T cells.^23^

## Results

### Characterisation of the TR repertoires

**Figure 1:**
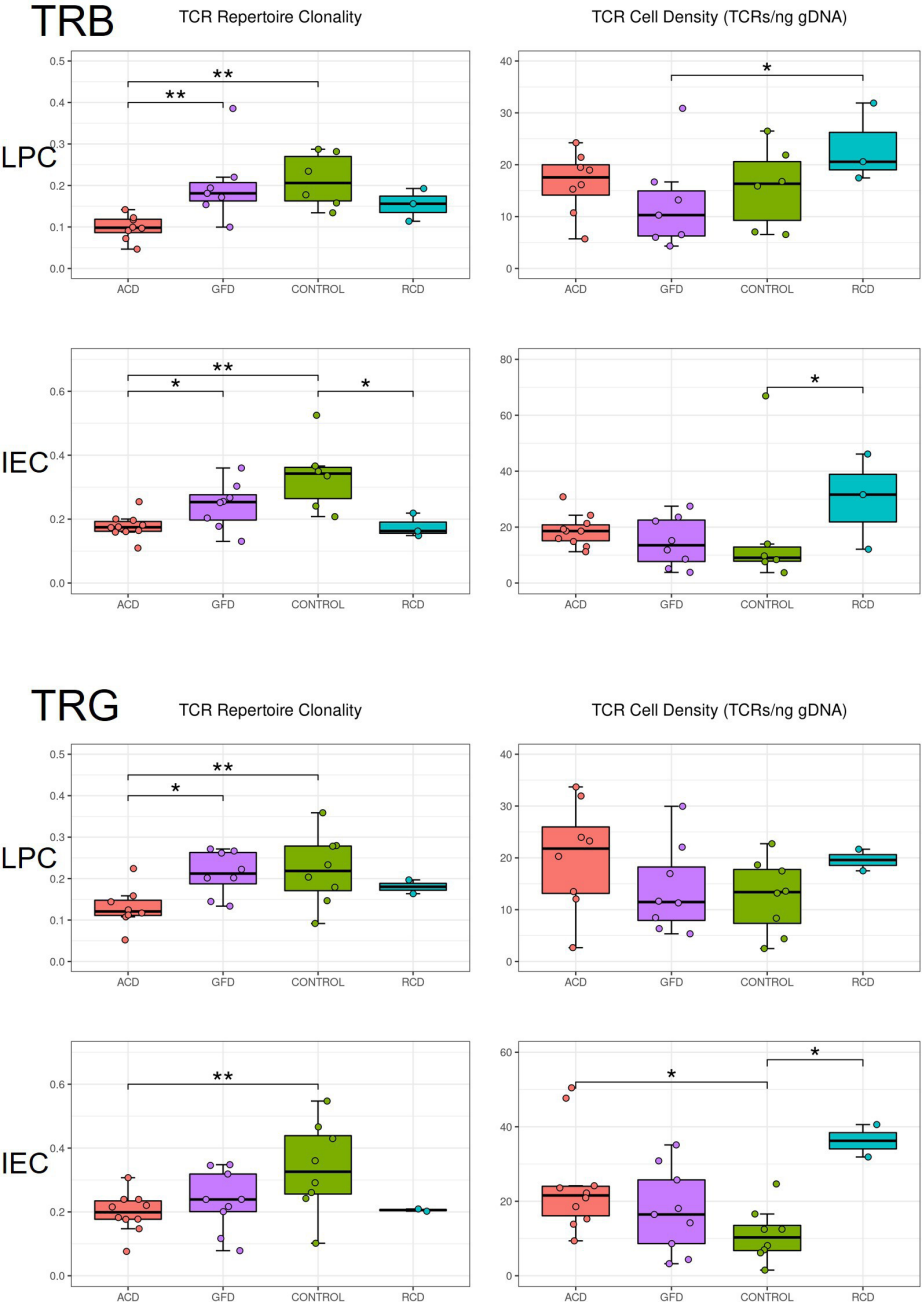
TR repertoire metrics by tissue type, TR locus and disease status. TR repertoire clonality (measured from 0 to 1) and T cell density (estimated TCRs per ng gDNA input) are charted by disease type. According to TR locus (TRB or TRG) and tissue cells (LPC or IEC) for each unique sample. Data are plotted with median and quartiles; significance is calculated using a Kruskal-Wallis test with a Dunn post-test, and significance is shown with bars and asterisks: (*p≤0.05; **p≤0.01). Jitter has been applied to the images to resolve closely spaced data elements. ACD, active coeliac disease; GFD, gluten-free coeliac disease; IEC, intraepithelial cell; LPS, lamina propria cell; RCD I, refractory coeliac disease type I.

The overall TR repertoire in each sample was characterised using two summary statistics: TR repertoire clonality, which is a normalised measure of the evenness of the TR frequency distribution,^24^ and T cell density (obtained by counting the number of observed TR molecules and dividing by the amount of genomic DNA used as input).^25^ Samples from 27 subjects were successfully sequenced for the TRB locus, including 24 paired, bulk (unsorted) IEC and LPC samples. Similarly, samples from 29 subjects were successfully sequenced for the TRG locus, including 25 with paired IEC and LPC bulk samples. Furthermore, six PBMC samples underwent successful sequencing of the TRB and TRG loci. Table 2 shows the mean productive nucleotide sequences by disease and sample type and figure 1 shows the distribution characteristics of the repertoire, separated by locus, tissue type and disease status. As shown in figure 1, the TR repertoire was fairly similar across loci and tissue types; among the 25 IEC/LPC pairs with informative TRG sequences and 24 IEC/LPC pairs with informative TRB sequences, both clonality and TR density were highly correlated between patients when comparing TRB vs TRG repertoires (Spearman’s r=X@0.75 and @0.81, respectively) and when comparing IEC vs LPC repertoires (Spearman’s r=X@0.70 and @0.71, respectively).

**Table 2:** Mean productive nucleotide sequences by disease and sample type

Patient status	Patients	Avg TRG productive sequences			Avg TRB productive sequences		
		IEC	LPC	PBMC	IEC	LPC	PBMC
Active coeliac disease	10	5469	7383	69 133	6249	8598	152 197
Refractory coeliac disease type 1	3	7954	8808	N/A	8570	11 608	N/A
Coeliac disease—gluten-free diet	9	5459	6677	46 601	3553	5570	106 076
Controls	8	3826	6006	55 286	1538	5259	146 125
**Total**	**30**						

Mean of the total number of nucleotide sequences with an AA product for each locus, disease state and sample origin are provided.

AA, amino acid; IEC, intraepithelial cell; LPC, lamina propria cell; N/A, not available; PBMC, peripheral blood mononuclear cell.

However, repertoire metrics differed across disease states; within each repertoire/locus/tissue combination. Figure 1 shows the results of a Kruskal-Wallis test with a post hoc Dunn test to determine significant differences between pairs of disease states. IEC and LPC samples (the two categories with the highest sample sizes) from control and ACD patients were significantly different in 6 of 8 comparisons, with the remaining two comparisons trending in the same direction: TRB and TRG repertoires of ACD IEC and LPC samples exhibited lower clonality than the repertoires of control samples, while there was a trend towards higher T cell density in ACD and RCD I samples compared with control and GFD samples. Interestingly, samples from subjects on a GFD had mean clonalities closer to control subjects than subjects with ACD (and to some extent RCD samples). The lower clonality seen in CD samples indicated that diseased tissue has a fairly broad and diverse TRB and TRG repertoire. The small number of PBMC samples did not demonstrate significant differences in clonality or T cell density between subjects (data not shown).

### Identification of CD-associated TR sequences in IEC samples

Public responses refer to identical, clonally dominant TRs shared by multiple individuals in a population^26^ and in the context of broad disease-associated TR repertoires, some public TRs may be more commonly encountered. To evaluate whether there were specific TRs associated with CD in our study cohort, we searched for public TRs (defined as CDR3 AA sequences present in at least 80% of unsorted samples) and tested for increased abundance in the diseased samples. There were no TRB AA sequences shared by more than 10 samples (37% of samples). Of 114 527 TRB AA sequences across all samples, 112 072 (97.9%) were unique to an individual sample. This distribution was similar between CD and control samples. In addition, there was no statistically significant difference in the relative V-gene utilisation in IEC samples from ACD, RCD I and GFD patients, or controls (Kruskal-Wallis rank sum test p=0.3122). These results indicate that the TRB repertoires of both patients with CD and individuals without CD are diverse and largely private, with the frequency of potential public TRs too low to be identified by our analytical approach.

**Figure 2:**
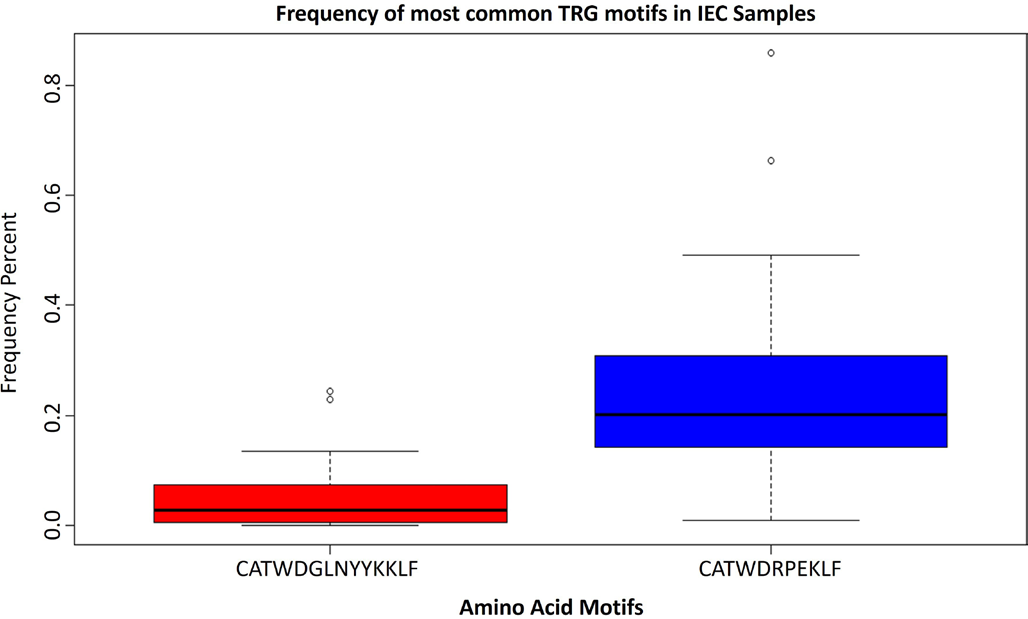
Relative frequency distribution of the two most abundant IE TRG CDR3 AA motifs identified in CD. A boxplot of the relative frequency distribution of the AA motifs CATWDRPEKLF and CATWDGLNYYKKLF in IEC samples from patients with CD shows that the CATWDRPEKLF AA sequence is considerably more abundant than CATWDGLNYYKKLF (Wilcoxon p=2.2e-05). AA, amino acid; CD, coeliac disease; IEC, intraepithelial cell.

On the contrary, 48 TRG CDR3 AA sequences were detected in 80% or more of the samples sequenced (online table S4). As a first screen, we performed Fisher’s exact test with a significance level of p<0.05 to identify sequences that were present more frequently in CD than in control repertoires, which revealed six different AA sequences. Among these, two sequences; CATWDGLNYYKKLF and CATWDRPEKLF, were significantly more abundant in CD samples versus controls as measured by a Mann-Whitney rank sum test with Bonferroni correction (p=0.0114 and 0.0136, respectively), which incorporated both presence and clonal abundance. We also compared the abundance of these sequences in ACD and GFD samples, with no significant differences noted (CATWDGLNYYKKLF p=0.6965, CATWDRPEKLF p=0.6334), indicating that the receptors with these AA motifs persist at comparable levels despite incorporation of GFD. When we compared the frequency distribution of these two TRG AA sequence motifs within the IEC samples from patients with CD, the shorter motif (CATWDRPEKLF), which was associated with the TRGV3 gene, had a significantly higher relative frequency distribution compared with CATWDGLNYYKKLF (figure 2).

10.1136/jcp-2022-208541.supp5 Supplementary data

### Identification of CD-associated TR sequences in LPC samples

There were no TRB AA sequences shared by more than 12 samples. Of 158 877 TRB AA sequences across all samples, 154 447 (97.2%) were unique to an individual sample. There was no statistically significant difference in the relative V-gene utilisation across LPC samples from ACD, RCD I and GFD patients, or controls (Kruskal-Wallis rank sum test p=0.9716). For TRG, 153 sequences were detected in 80% or more of the samples. No TRs were significantly more abundant in the LPC repertoires of patients with CD compared with controls. The longer sequence (CATWDGLNYYKKLF) was not observed in the LPC fractions of patients with CD, however, CATWDRPEKLF, the other high abundance AA motif detected in CD IEC samples, was also observed in LPC fractions of patients with CD but not in controls (Bonferroni adjusted p=0.063). Since the method used to separate IECs from LPCs does not yield cellular fractions of high purity, the detection of this motif in LPC samples could be due to the presence of residual IECs.

**Figure 3:**
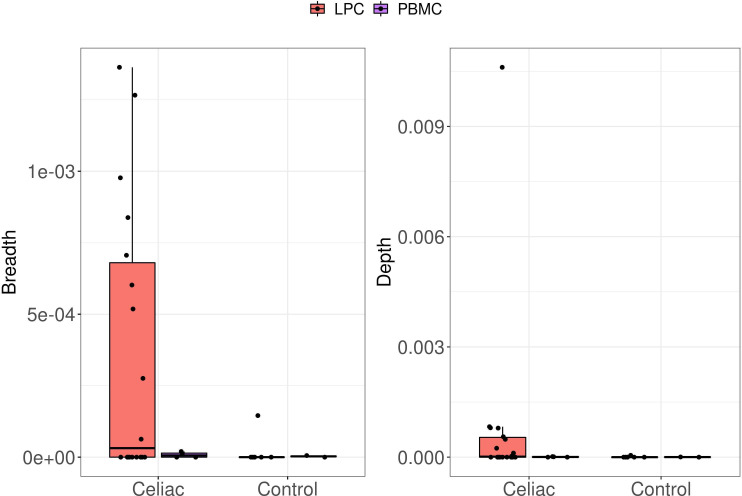
Detection of common gliadin-specific TRB sequences in CD samples. Plots depict comparison of the breadth (left) and depth of the gliadin-specific TCR sequences found within the aggregated (ACD, GFD, RCD) CD and control LPC and PBMC samples. Each LPC and PBMC sample was queried against a list of 218 published sequences. Breadth was calculated as the number of unique, matching, productive rearrangements out of the total number of unique productive rearrangements, while depth accounts for the frequency of those rearrangements in the repertoire. ACD, active CD; CD, coeliac disease; GFD, gluten-free diet; LPC, lamina propria cell; PBMC, peripheral blood mononuclear cell.

Although the full spectrum of gluten responsive T cell clonotypes is not known, prior TR sequencing studies of HLA class II tetramer-gliadin peptide sorted CD4^+^ T cells have identified public TR CDR3 sequences of HLA-DQ2.5 and DQ8 restricted gliadin-specific CD4^+^ T cells in a significant proportion of small intestinal LP and peripheral blood samples from patients with CD.^13 23^ To discern presence of gliadin-specific public TRB CDR3 sequences at a frequency below the stringent cut-off used in our study, we queried our bulk LPC (and PBMC) TRB CDR3 sequences against those generated in a recent large analysis of LP gliadin-specific CD4^+^ T cells isolated with HLA-DQ2.5-gliadin tetramers constituting four immunodominant epitopes.^23^ Nine of 18 (50%) of our CD LPC samples (3/8 ACD, 4/7 GFD and 2/3 RCD I) with successful TRB sequencing harboured CDR3 sequences similar to the published sequences, with an average of 4.9 shared (public) sequences per sample (range 1–10 per sample). In contrast, only 1 of 6 control LPC samples possessed a shared sequence with the published gliadin-specific CDR3 sequences, with only a single copy of the sequence being detected in that sample (figure 3). Public gliadin responsive TRB sequences were also detected in 2 of 4 PBMC samples from patients with CD, although at a relatively lower clonal breadth and depth. Among the 218 published gliadin-specific sequences, 23 were detected in our LPC and PBMC samples combined (details are provided in online table S3). Biased usage of specific TRBV genes by HLA-DQ2.5 and DQ8 restricted gliadin-specific CD4^+^ T cells has also been documented in CD.^13^ Since high-resolution HLA typing information was not available for our samples, as a proof of principle, we decided to query biased use of the TRBV7-2 gene, which has been observed in HLA-DQ2.5 restricted T cells. The TRBV7-2 gene was identified in 10/19 (53%) of the public TRB sequences in the 9 CD LPC samples, while the TRBV05-01 gene was noted in the only control sample with the single gliadin-specific public TRB sequence.

10.1136/jcp-2022-208541.supp4 Supplementary data

### Evaluation of sorted T cells from CD and control samples to determine the cellular source of TRG CDR3 motifs

As highlighted in table 1 and online table S1, in addition to sequencing of bulk samples, a few sorted IE CD8^+^ TCRαβ (n=8), IE TCRγδ (n=6) and LP CD4^+^ TCRαβ (n=6) T cell samples were subjected to sequencing of the TRG locus, in order to confirm IE TCRγδ T cells as the source of the TRG CDR3 sequences encoding the two AA motifs. The frequencies of the two motifs in unsorted samples were also compared with their frequencies in sorted samples. In these limited number of samples, the shorter motif (CATWDRPEKLF), in particular, was detected at a higher frequency in sorted IE TCRγδ T cell samples compared with matched unsorted samples; 1.8–2.5 fold increase for ACD samples and 3.1–4.5 fold increase for the GFD samples (online table S5). Online figure S1 depicts the relative frequency count for each motif across the sorted samples analysed. Importantly, these sequences were not detected in sorted IE CD8^+^ TCRαβ and LP CD4^+^ TCRαβ T cells.

10.1136/jcp-2022-208541.supp6 Supplementary data

### The TRG CDR3 motif CATWDRPEKLF is enriched in CD mucosa

Since the disease-associated increase in abundance of the two TRG CDR3 AA motifs in CD IECs compared with controls could be due to an increase in IE TCRγδ T cells that occurs in CD, we compared the frequency of the AA motifs in the blood and duodenal IECs/LPCs of patients with CD, using two ACD and two GFD donors for which matched samples were available. Intriguingly, among the four CD samples, the CATWDRPEKLF motif was on average enriched 12.1-fold (range 9.1–9.9 fold for ACD and 8.6–20.9 fold for GFD) in the IEC fractions (p<0.01), and it was also enriched in the LPC samples. However, such differences were not observed for the longer AA motif (CATWDGLNYYKLF).

### Distribution and HLA association of the putative CD-associated TRG CDR3 motif CATWDRPEKLF

**Figure 4:**
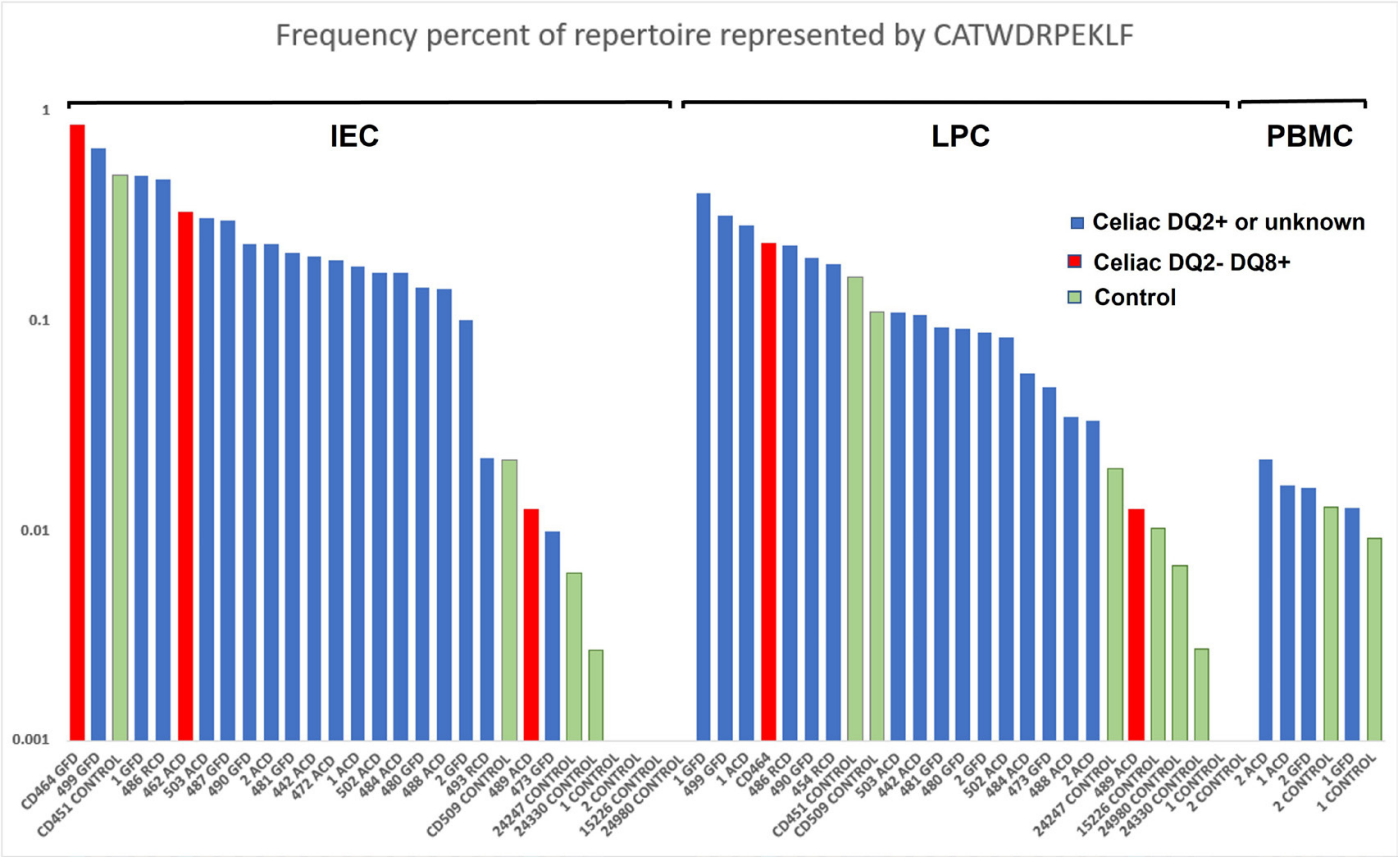
Abundance of CATWDRPEKLF in the study cohort. The frequency of the AA motif CATWDRPEKLF across all samples (logarithmic scale). The samples were separated according to origin; duodenal biopsy intraepithelial cells (IEC) or lamina propria cells (LPC) fractions or peripheral blood mononuclear cell (PBMC) samples. Patients with CD who were HLA DQ2^+^ or of unknown HLA type were distinguished from the patients who were HLA DQ2^neg^DQ8^+^. Patients without CD were designated as controls. AA, amino acid; CD, coeliac disease.

At least 90% of patients with CD have the HLA-DQ2 risk allele, with the majority of the remainder carrying the HLA-DQ8 allele.^27^ To assess any relationship between the patient HLA type and the putative CD-associated TRG CDR3 AA motif, we calculated the repertoire frequency of the motif in each sample and plotted the results in descending order of frequency based on sample types (IECs, LPCs and PBMCs). CD samples were grouped as HLA-DQ2^neg^DQ8^+^, HLA-DQ2^+^/HLA unknown and controls (figure 4). The receptor motif CATWDRPEKLF was relatively evenly distributed between the DQ2^neg^DQ8^+^ and DQ2^+^/HLA unknown samples, consistent with absence of HLA-DQ2 or DQ8 restriction of the motif bearing TCRγδ T cells.^6^ Every sample with a CD diagnosis harboured this TR sequence motif, as did 12 of 18 control samples (inclusive of sorted TCRγδ IEC samples). Abundance was high in most IEC and LPC samples from both HLA-DQ2^+^/HLA unknown and DQ2^neg^DQ8^+^ CD patients and low in most control samples.

### Evaluation of TR rearrangements constituting the TRG CDR3 motif CATWDRPEKLF

We next sought to determine if the abundant and tissue enriched TRG CDR3 AA motif CATWDRPEKLF in patients with CD was derived from unique clonal sequences in each sample or was the consequence of several TRG rearrangements with different sequences all leading to the same TRG CDR3 AA motif by convergent recombination. The constituent nucleotide sequences encoding the CDR3 AA motif CATWDRPEKLF in each sample were assessed. Table 3 shows the average number of unique CDR3 nucleotide variants in each sample, by disease status and sample type. In contrast to control tissue samples, CD IEC and LPC samples had a mean average of ~23 separate nucleotide sequences (different TRG rearrangement events) encoding the TRG.CDR3 AA motif CATWDRPEKLF, demonstrating that the motif is recurrently produced during V-J recombination, and suggesting that several clonal lineages bearing the same TRG CDR3 sequence at the AA level are present in CD mucosa. As for PBMCs samples, an average of 17 distinct rearrangements (range 9–25) generated this motif in ACD, with no significant differences between CD subjects and controls overall. A small number of rearrangements common to both IEC and PBMC samples from the same subject were present at comparable levels to the same rearrangements between samples from unrelated subjects, indicative of a high generation probability for these rearrangements.

**Table 3:** Unique nucleotide variants encoding CATWDRPEKLF in CD and control samples

CATWDRPEKLF	IEC		LPC		PBMC	
	Average	Range	Average	Range	Average	Range
ACD	21.7	5–42	14.1	1–33	17.0	9–25
GFD	20.9	2–62	19.6	8–46	11.5	11–12
RCD I	27	10–44	34	34		
Control	<1*	0–1	2	1–3	14.0	11–17

The mean and range of unique CDR3 nucleotide variants encoding the CDR3 region CATWDRPEKLF AA motif is shown for CD patient and control IEC, LPC and PBMC samples.

*Absolute value in decimals not shown due to lack of biological meaning for values below 1.

ACD, active CD; CD, coeliac disease; GFD, gluten-free diet; IEC, intraepithelial cell; LPC, lamina propria cell; PBMC, peripheral blood mononuclear cell; RCD, refractory CD.

## Discussion

In the current study, high throughput TR sequencing analysis of small intestinal IEC and LPC samples from CD patients, including those with ACD and RCD I as well as individuals on GFD, and non-CD controls, revealed significantly lower TRB and TRG clonality in CD compared to controls, indicative of increased diversity of the T cell repertoires in CD. Our findings differ from those of an earlier study that reported an oligoclonal IEL TRB repertoire in CD,^14^ but corroborate the results of recent studies analysing small intestinal TR profiles of CD patients with different clinical presentations and utilising different assays, including PCR using the BIOMED II primers,^28^ multiplex PCR followed by high throughput sequencing,^29^ and single cell sequencing of TCRαβ CD8^+^ and TCRγδ IELs.^30 31^ Our sequencing and analytical approach demonstrated the small intestinal IE and LP TRB repertoires in CD to be unique overall, showing low overlap between samples and minimal publicity of the sequences, without differential abundance of any CDR3 AA motif in CD compared with controls. However, comparison of LP (and PBMC) TRB CDR3 sequences with published sequences derived from HLA-DQ2.5 gliadin tetramer-sorted LP CD4^+^ T cells,^23^ disclosed the presence of gliadin-specific public TRB sequence in 50% of our CD samples, at levels below the detection threshold employed in our study, and also biased TRBV7-2 gene usage. On the other hand, despite the increased diversity of the TRG repertoire in CD, we observed enrichment of certain TCRγδ T cell derived TRG CDR3 sequence motifs in the small intestinal mucosa of CD patients, which has not been reported before.

As it is well established that the adaptive immune response to gluten-derived deamidated gliadin peptides is the prime driver of mucosal inflammation in CD, most of the studies to date have focused on characterising the repertoire, phenotype and functional attributes of gliadin-specific CD4^+^ T cells within the small intestinal LP (and peripheral blood).^13^ However, due to the rarity of gliadin responsive T cells in the LP of CD patients (<2% of T cells),^32^ soluble HLA-DQ multimers bound with gliadin peptides and other enrichment strategies have been utilised to isolate CD4^+^ T cells that recognise immunodominant epitopes of gliadin peptides presented by HLA-DQ2 and DQ8 molecules.^33–37^ The use of pooled HLA-DQ2 gliadin tetramers has helped delineate the breadth of gluten-specific T cell responses in CD.^23 38 39^ These studies have revealed unique characteristics of the gluten responsive CD4^+^ TR repertoires in CD, including occurrence of gliadin-specific public TRs in a high proportion of cases (94%), although with limited overlap of the TRs (2–23 shared sequences) among patients, biased or preferential use of certain TRAV, TRBV and TRAV/TRBV pairs, and conserved CDR3 motifs. Our observation of a diverse, polyclonal TRB repertoire in the small intestinal LP in ACD and RCD I suggests similarities between these two disease states, and indicates a preponderance of gluten non-specific T cells in the inflamed LP that traffic to this site because of the altered cytokine milieu and impaired intestinal barrier function engendered by ongoing adaptive and innate mucosal immune responses. Dampening of inflammation on commencement of GFD leads to a shift in the T cell repertoire towards the normal state with increased TRB clonality.^40^ The prevalence of gliadin responsive CD4^+^ T cells has also been shown to be higher in the inflamed CD mucosa, which decrease on removal of gluten from the diet, but persist at low levels for long periods.^39^ Since the goal of our study was to detect high abundance disease-specific public sequences that could be exploited for diagnostic purposes, our analysis pipeline was unable to identify low frequency gliadin-specific public TRB sequences in an unbiased manner.

Dysregulated IL-15 expression by epithelial cells and LP macrophages incited by toxic gliadin peptides and cytokines elaborated by gliadin responsive LP CD4^+^ T cells (eg, IL-2, IL-21, TNFα) have been proposed to activate innate CD8^+^ TCRαβ IELs that kill epithelial cells, which have upregulated ‘stress-induced’ ligands, in a TCR-independent fashion.^41–43^ Although clonal expansions of circulating gut homing CD8^+^ TCRαβ T cells with preferential usage of specific TRBV genes was reported after gluten challenge in CD patients,^44^ the observation of a more diverse IE TRB repertoire by us, and CD8^+^ TRA/B repertoire by others,^31^ in ACD compared with GFD and control samples, and the absence of TRBV gene bias, supports the polyclonal nature of the IE CD8^+^ αβ T cell immune response in CD. This was also true for RCD I, a subtype of RCD that is characterised by a polyclonal expansion of IE CD8^+^ TCRαβ T cells, as opposed to RCD II, which represents a clonal lymphoproliferative disorder of IELs manifesting an aberrant immunophenotype.^45^ The detection of lower clonality and higher T cell density in RCD I, ascertained by TRB (and TRG) sequencing, indicates persistence of an inflammatory process that is similar in nature to ACD, in line with previous findings.^29^

TCRγδ IELs are elevated in CD, but the roles of these lymphocytes in disease pathogenesis remains unclear. Besides an immunoregulatory role proposed for a subset,^4^ they likely also subserve other functions. Mayassi *et al* reported loss of naturally occurring Vγ4^+^/Vδ1^+^ TCRγδ IELs in ACD that have cytocidal properties and recognise epithelial butyrophilin-like (BTNL) molecules (BTNL3/BTNL8), reduced epithelial BTNL8 expression and a concomitant expansion of gluten sensitive IFN-γ producing TCRγδ Vδ1^+^ IELs whose ligands are unknown.^46^ An expansion of circulating TCRγδ T cells, with preferential usage of the TRDV1 gene has also been described in CD patients after gluten challenge.^44^ In contrast, recent single cell sequencing analysis of TCRγδ IELs and gut-homing TCRγδ T cells in the peripheral blood by Eggesbø *et al* demonstrated biased usage of TRGV4 and TRDV1 genes in healthy individuals, but diverse TCRγδ T cell repertoires in samples from ACD patients and those on GFD.^30^ We also observed increased diversity of the IE TRG repertoires in CD, however higher abundance of a couple of public TRG CDR3 AA sequences was noted in samples from CD patients, including those on GFD, compared with controls. These sequences were confirmed to be derived from TCRγδ T cells and not from companion TRG rearrangements in TCRαβ T cells. One of the CDR3 AA motifs, CATWDRPEKLF, associated with the TRGV3 gene, was enriched in IECs compared with matched PBMC samples. Evaluation of the CDR3 nucleotide sequences revealed that the AA sequence was generated from multiple nucleotide variants, suggesting convergent evolution. Interestingly, many of the gliadin-specific public TRB CDR3 AA sequences reported previously also resulted from convergent recombination.^39^

Although Eggesbø *et al* detected several public TRG CDR3 AA sequences in small intestinal TCRγδ IELs of CD patients, they did not identify significant enrichment of any sequence in CD samples.^30^ It is unclear if this was due to a lower number of productive TRG sequences assessed by the authors. Of interest, using bulk sequencing of DNA extracted from duodenal biopsies and machine learning analysis, Foers *et al*, recently reported CD-associated changes in TRG and TRD repertoires, which persisted even after cessation of gluten consumption.^15^ It is possible that restricted diversity of the TRG repertoire enabled detection of the TRG AA motifs in CD samples. The significance of enrichment of CATWDRPEKLF motif-bearing TCRγδ T cells in the small intestinal epithelium of CD patients, however, remains to be determined. We speculate that TCRγδ T cells bearing this TR motif traffic to and accumulate (or proliferate) in the small intestinal epithelium in response to epithelial damage or stress induced by toxic gliadin peptides, since complete exclusion of dietary gluten can be difficult for CD patients. Paired TRG and TRD sequencing might enable better characterisation of such public TR bearing TCRγδ T cells in CD and help ascertain similarities or differences between our findings and those published previously. If confirmed by others, the identified TRG CDR3 motif could serve as a novel biomarker of CD.

A limitation of the methodology used in this study is that it is unsuitable for detecting gliadin-specific T cells in an unbiased manner due to: scarcity of these T cells in the small intestinal mucosa (and peripheral blood), limited sharing of public TR sequences among patients, and incomplete knowledge of the breadth of gliadin epitopes in CD.

In summary, NGS-based analysis of the TR repertoires of small intestinal IEC and LPC samples revealed reduced clonality of TRB and TRG repertoires in CD, suggesting recruitment of diverse populations of TCRαβ and TCRγδ T cells to the inflamed small intestinal mucosa. An increased abundance of IE TCRγδ T cells bearing specific public TRG CDR3 sequences was also noted in CD. Future studies, utilising single cell TR sequencing coupled with immunophenotypic and transcriptional analyses,^47^ are required to elucidate the biologic attributes of these and other mucosal T cell populations in CD.

## Data Availability

Data are available in a public, open access repository.
