# Supplementary material for: Characterisation of T cell receptor repertoires in coeliac disease
Source: J Clin Pathol. 2022 Dec 15;77(2):116–24. doi: 10.1136/jcp-2022-208541 (PMC10850686; doi:10.1136/jcp-2022-208541)
Supplement: Supplementary data [file jcp-2022-208541supp001.pdf]

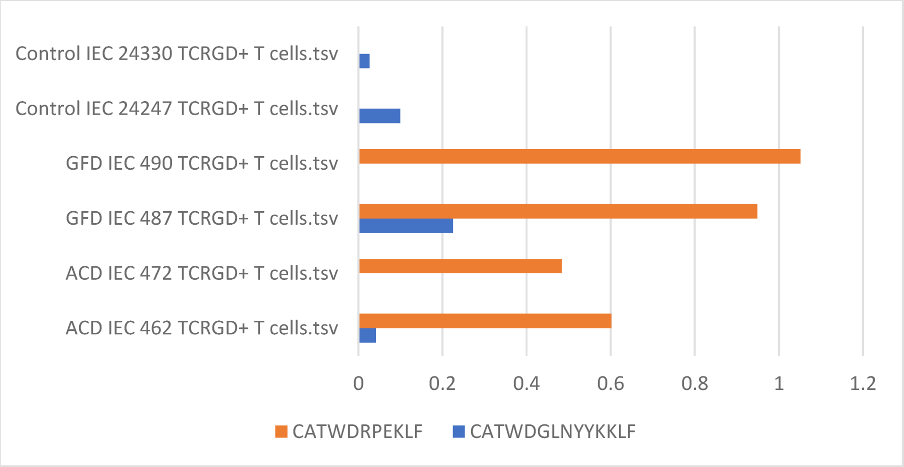

v

Figure S1. Relative frequency count of the two most significant TRG AA motifs within the sorted IEC TCRγδ T cells of CD patients and controls. Bar plot of the relative frequency count (sum across sequences belonging to a sample) of the AA motifs CATWDRPEKLF and CATWDGLNYYKKLF in IEC samples from CD patients shows the higher abundance of CATWDRPEKLF AA sequence and its absence in control samples.

SUPPLEMENTAL METHODS

Cell isolation

Fresh biopsy specimens were put in RPMI solution containing 1mM eEGTA (VWR), 1.5 mM $MgCl_2$ (Sigma Aldrich, St. Louis, USA) and 1% FBS (Mediatech, Virginia, USA) to isolate IECs. After stirring for one hour at 37°C, cell suspensions were pelleted from the supernatant and washed two times in culture media containing RPMI (Mediatech) supplemented with 10% FBS, 10,000 IU penicillin and 10,000 μg/mL streptomycin. The remaining tissue sample was incubated in collagenase to release the LPCs.

For PBMC isolation, equal amounts of PBS and blood (7-8 mL/sample) were added to a 50 mL tube and mixed, following which the mixture was layered over an equal volume of Ficoll-Hypaque (MilliporeSigma) using an automatic pipette and centrifuged at 1200 rpm for 20 minutes at 4°C. The interface between the plasma and PBS/Ficoll-Hypaque was transferred to a new 15 mL tube and centrifuged at 1300 rpm for 10 minutes. The pellet was reconstituted in 0.5 mL of FBS. After counting the cells, aliquots of 1 million cells were suspended in DMSO/FBS and stored in liquid nitrogen.

CD patient categorization

CD patients were separated into categories based on disease status: newly diagnosed or active celiac disease (ACD), refractory celiac disease type 1 (RCD I, no response to the gluten-free diet for >12 months, with the IECs manifesting a normal immunophenotype) and CD controlled by a gluten-free diet (GFD). All 30 patients within the study were subjected to endoscopy and small intestinal biopsy. The clinical, histopathologic and laboratory data of all patients are provided in Table S1.

Cell-sorting strategy

The IECs and LPCs were stained with CD45 A700, CD3 PE, CD4 APC-Cy7, CD8 FITC, TCRβ PE-Cy7 and TCRγ BV421. After gating on CD3/CD45 double positive events, a bivariate plot was created looking at TCRβ and TCRγ expression in IEC samples. TCRγδ T cells were sorted by gating on the $TCR\gamma^{+}$ $TCR\beta^{-}$ population. To identify TCRαβ IEC and LPC populations of interest, after gating on $TCR\beta^{+}$ events, a bivariate plot was created for CD8 and CD4 expression, and TCRβ-gated $CD8^{+}$ and $CD4^{+}$ T cells were sorted from IEC and LPC samples, respectively.

TR sequencing approach

Briefly, a multiplexed PCR method that uses a mixture of forward primers specific to V gene segments and reverse primers specific to J gene segments was employed. Sequencing of amplified CDR3 regions was performed using the Illumina HiSeq System (Illumina Inc., San Diego, USA). Raw HiSeq sequence data were preprocessed to remove errors in the primary sequence of each read. To remove both PCR and sequencing errors, a nearest neighbor algorithm was used to collapse the data into unique sequences by merging closely related sequences[16,17].
