# Supplementary material for: Characterisation of T cell receptor repertoires in coeliac disease
Source: J Clin Pathol. 2022 Dec 15;77(2):116–24. doi: 10.1136/jcp-2022-208541 (PMC10850686; doi:10.1136/jcp-2022-208541)
Supplement: Supplementary data [file jcp-2022-208541supp003.pdf]

Subject ID	Disease status	Sample type	T cell lineage sorted	Number of events/cells sorted
24330	Control	IEC	CD8 TCRαβ	100,000
24247	Control	IEC	CD8 TCRαβ	3683
488	ACD	IEC	CD8 TCRαβ	26523
489	ACD	IEC	CD8 TCRαβ	12646
472	ACD	IEC	CD8 TCRαβ	143000
462	ACD	IEC	CD8 TCRαβ	167000
490	GFD	IEC	CD8 TCRαβ	9575
487	GFD	IEC	CD8 TCRαβ	168000
Subject ID	Disease status	Sample type	T cell lineage sorted	Number of events/cells sorted
24330	Control	LPC	CD4 TCRαβ	204036
24247	Control	LPC	CD4 TCRαβ	9836
488	ACD	LPC	CD4 TCRαβ	5693
489	ACD	LPC	CD4 TCRαβ	8058
490	GFD	LPC	CD4 TCRαβ	2452
473	GFD	LPC	CD4 TCRαβ	11043
Subject ID	Disease status	Sample type	T cell lineage sorted	Number of events/cells sorted
24330	Control	IEC	TCRγδ	78294
24247	Control	IEC	TCRγδ	133000
472	ACD	IEC	TCRγδ	3400
462	ACD	IEC	TCRγδ	176000
487	GFD	IEC	TCRγδ	177000
490	GFD	IEC	TCRγδ	6600
