# Supplementary material for: Characterisation of T cell receptor repertoires in coeliac disease
Source: J Clin Pathol. 2022 Dec 15;77(2):116–24. doi: 10.1136/jcp-2022-208541 (PMC10850686; doi:10.1136/jcp-2022-208541)
Supplement: Supplementary data [file jcp-2022-208541supp006.pdf]

Sample ID	CATWDRPEKLF Frequency
ACD_IE_462_TCRGD__T_CELLS	0.601
ACD_IE_462_UNSORTED_IEs	0.329
ACD_IE_472_TCRGD__T_CELLS	0.484
ACD_IE_472_UNSORTED_IEs	0.194
GFD_IE_487_TCRGD__T_CELLS	0.948
GFD_IE_487_UNSORTED_IEs	0.301
GFD_IE_490_TCRGD__T_CELLS	1.051
GFD_IE_490_UNSORTED IEs	0.232
Control_IE_24247_TCRGD__T_CELLS	0.000
Control_IE_24247_UNSORTED_IEs	0.003
Control_IE_24330_TCRGD__T_CELLS	0.000
Control_IE_24330_UNSORTED_IEs	0.012

Sample ID	CATWDGLNYYKKLF Frequency
ACD_IE_462_TCRGD__T_CELLS	0.042
ACD_IE_462_UNSORTED_IEC	0.003
ACD_IE_472_TCRGD__T_CELLS	0.000
ACD_IE_472_UNSORTED_IEC	0.012
GFD_IE_487_TCRGD__T_CELLS	0.225
GFD_IE_487_UNSORTED_IEC	0.228
GFD_IE_490_TCRGD__T_CELLS	0.000
GFD_IE_490_UNSORTED_IEC	0.021
Control_IE_24247_TCRGD__T_CELLS	0.099
Control_IE_24247_UNSORTED_IEC	0.024
Control_IE_24330_TCRGD__T_CELLS	0.026
Control_IE_24330_UNSORTED_IEC	0.012
